# A Designed Angiopoietin-1 Variant, Dimeric CMP-Ang1 Activates Tie2 and Stimulates Angiogenesis and Vascular Stabilization in N-glycan Dependent Manner

**DOI:** 10.1038/srep15291

**Published:** 2015-10-19

**Authors:** Nuri Oh, Kangsan Kim, Soo Jin Kim, Intae Park, Jung-Eun Lee, Young Suk Seo, Hyun Joo An, Ho Min Kim, Gou Young Koh

**Affiliations:** 1 Graduate School of Medical Science and Engineering, Korea Advanced Institute of Science and Technology (KAIST), Daejeon, 305-701, Korea; 2 Department of Biological Sciences, Korea Advanced Institute of Science and Technology (KAIST), Daejeon, 305-701, Korea; 3 Graduate School of Analytical Science and Technology, Chungnam National University, Daejeon, 305-764, Korea

## Abstract

Angiopoietin-1 (Ang1), a potential growth factor for therapeutic angiogenesis and vascular stabilization, is known to specifically cluster and activate Tie2 in high oligomeric forms, which is a unique and essential process in this ligand-receptor interaction. However, highly oligomeric native Ang1 and Ang1 variants are difficult to produce, purify, and store in a stable and active form. To overcome these limitations, we developed a simple and active dimeric CMP-Ang1 by replacing the N-terminal of native Ang1 with the coiled-coil domain of cartilage matrix protein (CMP) bearing mutations in its cysteine residues. This dimeric CMP-Ang1 effectively increased the migration, survival, and tube formation of endothelial cells via Tie2 activation. Furthermore, dimeric CMP-Ang1 induced angiogenesis and suppressed vascular leakage *in vivo*. Despite its dimeric structure, the potencies of such Tie2-activation-induced effects were comparable to those of a previously engineered protein, COMP-Ang1. We also revealed that these effects of dimeric CMP-Ang1 were affected by specified N-glycosylation in its fibrinogen-like domain. Taken together, our results indicate that dimeric CMP-Ang1 is capable of activating Tie2 and stimulating angiogenesis in N-glycan dependent manner.

Angiopoietin-1 (Ang1) is a secretory ligand of tyrosine kinase with Immunoglobulin and epidermal growth factor homology domain 2 (Tie2) that is mainly expressed by vascular endothelial cells and hematopoietic cells^1^,^2^,^3^. Ang1 plays critical roles in vascular assembly, maturation and stabilization, coronary venogenesis, and glomerular vascular protection during developmental and pathological angiogenesis^2^,^3^,^4^,^5^,^6^,^7^. The structure of Ang1 consists of a carboxyl-terminal fibrinogen-like domain (FLD) that binds to the Tie2 receptor, a central coiled-coil domain that oligomerizes the fibrinogen-like domains, and a short amino-terminal domain that superclusters the oligomer into variably sized multimers^8^,^9^,^10^. In fact, Ang1 naturally exists as heterogeneous oligomers with trimer, tetramer, and higher-order oligomers^9^,^10^,^11^. Due to such inert nature of Ang1, recombinant Ang1 that is produced in mammalian cells is non-soluble, sticky, non-specifically bondable, and easily aggregative^11^,^12^. These unavoidable features of recombinant Ang1 leads to difficulty in producing as a therapeutic protein in mass as Tie2 activator.

Oligomeric structure of Ang1 is required for the clustering of Tie2^2^,^3^,^9^,^10^,^11^,^13^, leading to the phosphorylation of Tie2 kinase domain and initiation of downstream signaling pathways. This Ang1-induced Tie2 clustering could be a unique and essential process for efficient signaling, unlike any other receptor tyrosine kinases^14^,^15^. Tie2 recognition and binding is predominantly mediated by the fibrinogen-like domain of Ang1. However, the fibrinogen-like domain alone is not sufficient for the activation of Tie2. Furthermore, biochemical studies demonstrated that at least trimeric Ang1 is required for the activation of Tie2 receptor, and recombinant dimeric Ang1 proteins, GCN-Ang1 and Ang1-F1-Fc, were unable to activate Tie2 receptor^8^,^9^,^11^. Thus, the oligomeric status of Ang1 was readily accepted as a critical determinant for the activation of Tie2. Based on this unique interaction between secretory ligand and membrane receptor, our group previously generated a soluble, stable, and potent pentameric Ang1 variant by replacing the N-terminal domain of Ang1 (245 amino acids) with the short, pentameric coiled-coil domain (45 amino acids) of the cartilage oligomeric matrix protein (COMP), and named it as “COMP-Ang1”^11^. Thus far, COMP-Ang1 has been extensively used in numerous experimental settings as an alternative to native Ang1, and has provided adequate rationales and insights regarding the importance of vascular regeneration^3^. However, COMP-Ang1 is a large protein (~175 kDa) that is difficult to produce, purify, and store in a stable form, posing unavoidable barriers to be developed as a therapeutic protein. Therefore, substantial advances are required to develop a simple, small, and potent Ang1 variant for therapeutic purposes.

In this study, we generated a simple and potent Ang1 variant (CMP-Ang1) by replacing the coiled-coil domain of native Ang1 with the short, trimeric coiled-coil domain of cartilage matrix protein (CMP). We then modified the cysteines within the coiled-coil and linker domains using site-directed mutagenesis to create a dimeric form of Ang1, named CMP-A1-3. Unlike other previously developed dimeric Ang1 variants, CMP-A1-3 was able to activate Tie2 and its downstream signaling, which are dependent on N-glycosylation and the type of the attached glycan. Of special note, CMP-A1-3 promotes angiogenesis in a Tie2-dependent manner to a similar level to that of COMP-Ang1. Taken together, our findings show that dimeric CMP-Ang1 is a potent Ang1 variant that activates Tie2 receptor and stimulates angiogenesis, and hence could be developed as a therapeutic protein.

## Results

### Design and characterization of CMP-Ang1 variants

**Figure 1:**
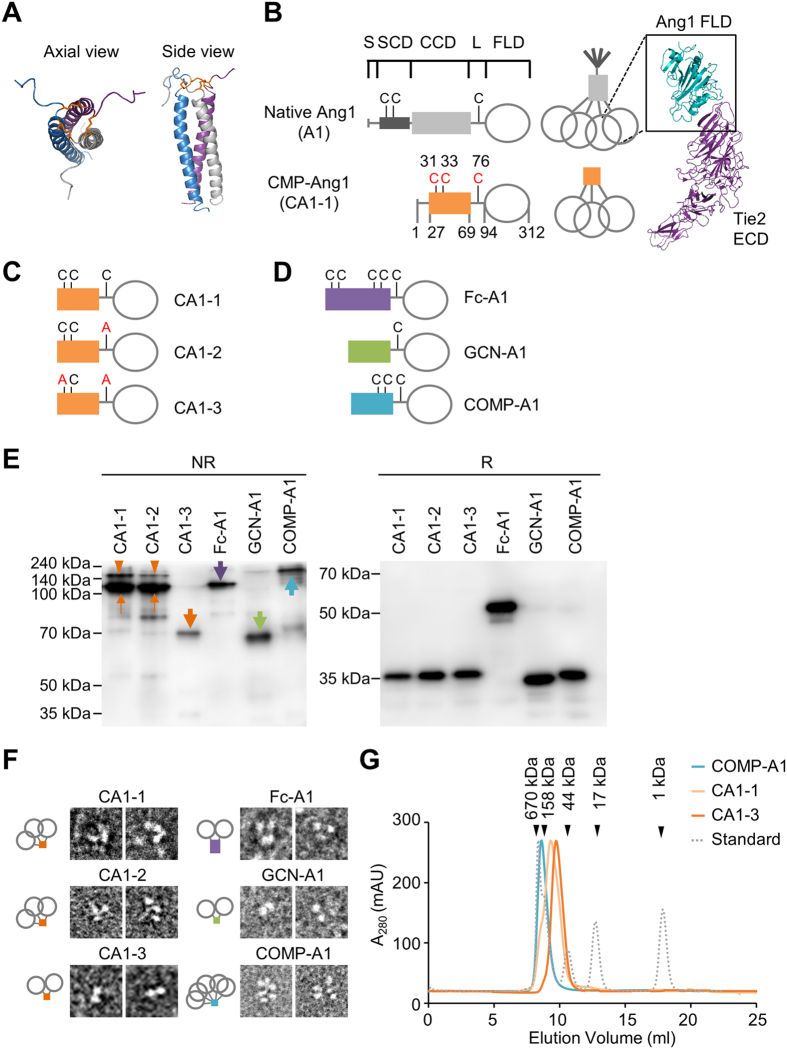
Design and characterization of CMP-Ang1 and its variants. (**A**) Axial and side views of CMP structure (PDB: 1AQ5). Coiled-coil domain of CMP are trimerized and stabilized by disulfide bonds. Orange, the three disulfide bonds. (**B**) Schematic diagram of native Ang1 and CMP-Ang1. Ang1 consists of the secretory signal sequences (S), super-clustering domain (SCD), coiled-coil domain (CCD), linker (L), and fibrinogen-like domain (FLD). Crystal structure of Ang1-Tie2 complex (PDB: 4K0V) indicates that FLD of Ang1 is essential to bind to Tie2 receptor. Bluish green, FLD of Ang1; purple, extracellular domain (ECD) of Tie2. The SCD and CCD of Ang1 are replaced with the short CCD of CMP to generate CMP-Ang1 (denoted CA1-1). (**C**) Schematic diagrams of CMP-Ang1 variants. In CA1-2, cysteine 76 is replaced with alanine; in CA1-3, cysteines 31 and 76 are replaced with alanine. (**D**) For hFc-Ang1, GCN-Ang1, and COMP-Ang1, the SCD and CCD of Ang1 are replaced with the Fc portion of human IgG1 and with the short coiled-coil domains of GCN and COMP, respectively. (**E**) Ang1 variants were separated by SDS-PAGE under non-reduced (NR) and reduced (R) conditions, and were immunoblotted with anti-Ang1 antibody. In NR condition, CA1-1 and CA1-2 mainly form trimers (orange thin arrows) but slightly exist as higher order oligomers (orange arrowheads), while CA1-3, GCN-Ang1, Fc-Ang1, and COMP-Ang1 mostly form dimers, dimers, dimers, and pentamers, respectively (thick arrow of each different color). Molecular masses (kDa) are indicated on the left. (**F**) The oligomeric states of recombinant proteins were imaged by electron microscopy; respective schematic depictions are shown below. CA1-1 and CA1-2 mainly form trimers, whereas CA1-3, GCN-Ang1, and Fc-Ang1 form dimeric structures. COMP-Ang1 shows pentameric structures. (**G**) Oligomeric statuses of COMP-Ang1, CA1-1 and CA1-3 were analyzed using a size-exclusion chromatography. The peaks for COMP-Ang1, CA1-1 and CA1-3 correspond to pentamer (~175 kDa), trimer (~105 kDa) and dimer (~70 kDa), respectively. Arrowheads indicate the molecular masses of standards.

As depicted in Fig. 1A, human CMP contains a short coiled-coil domain (43 amino acids; EEDPCACESLVKFQAKVEGLLQALTRKLEAVSKRLAILENTVV) that functions as a trimer assembly that is stabilized by forming three inter-chain disulfide bonds^16^,^17^. Using this trimer assembly, we designed trimeric Ang1, namely CMP-Ang1-1 (CA1-1), in which the N-terminal super-clustering domain and coiled-coil domain of human Ang1 (245 amino acid) were replaced with the short trimeric CMP (Fig. 1B). Then, CA1-1 was generated by a transient mammalian expression system using HEK293 cells^10^,^18^. SDS-PAGE analysis revealed that recombinant CA1-1 protein mostly formed ~105 kDa trimers but also scarcely existed as ~70 kDa dimer and ≥200 kDa higher order oligomers under NR condition, while it mainly formed ~35 kDa monomer under R condition (Fig. 1E).

Because cysteine residues are essential for the disulfide bonds, and these disulfide bonds have critical roles in maintaining oligomeric structures, we sought to test how the cysteine residues in the linker of Ang1 and in the coiled-coil domain of CMP affect the oligomeric status of CMP-Ang1 by replacing them with alanine with different combinations (Fig. 1C), and the newly created CMP-Ang1 variants were generated and analyzed using the same methods. As controls, we also generated Fc-Ang1 (Fc-A1), GCN-Ang1 (GCN-A1) and COMP-Ang1 (COMP-A1) according to previous reports^9^,^11^ using the same methods (Fig. 1D). Interestingly, the replacement of cysteine with alanine in the linker (CMP-Ang1-2; CA1-2) showed a similar oligomeric pattern with CA1-1, while the replacement of the first cysteine in the coiled-coil domain and cysteine in the linker with alanine (CA1-3) led to the formation of solely dimeric Ang1 (~70 kDa) under NR condition (Fig. 1E). All recombinant proteins showed expected sizes of monomers in R condition (Fig. 1E). Further TEM analysis also indicated that CA1-1 and CA1-2 mainly formed trimers, whereas CA1-3 only formed dimers without higher ordered oligomers (Fig. 1F). In comparison, SDS-PAGE and TEM analysis showed that Fc-Ang1, GCN-Ang1, and COMP-Ang1 mainly formed dimers, dimers, and pentamers, respectively, which is consistent with previous findings^9^,^13^,^15^ (Fig. 1E,F). Furthermore, we performed a size-exclusion chromatographic analysis for COMP-Ang1, CA1-1 and CA1-3 to clearly verify their oligomeric statuses. Distinct peaks corresponding pentameric COMP-Ang1 (~175 kDa), trimeric CA1-1 (~105 kDa) and dimeric CA1-3 (~70 kDa) were shown (Fig. 1G).

We then analyzed the biochemical characteristics of these recombinant proteins. The recombinant proteins were secreted from HEK293 cells and bound to their respective purification matrix, streptactin agarose beads. Approximately 85% of most recombinant proteins were recovered from the purification matrix, while only ~65% of native Ang1 was recovered from the purification matrix (Table 1). A solubility assay with acid precipitation indicated that CMP-Ang1 variants, Fc-Ang1, GCN-Ang1, and COMP-Ang1, were relatively soluble, while native Ang1 had low solubility (Table 1). Furthermore, CA1-1, CA1-3 and COMP-Ang1 maintained their Tie2 phosphorylation activity, while that of native Ang1 was lost by approximately 40% (Table 1). Importantly, unnoticeable aggregation was detected in CMP-Ang1 variants, while a notable aggregation was detected in ~10% of COMP-Ang1 and ~50% of native Ang1 (Table 1).

Thus, we were able to obtain soluble, stable, and low molecular-sized, trimeric and dimeric Ang1 variants by replacing the N-terminal of Ang1 with the short coiled-coil domain of CMP and modifying the inter-chain disulfide cysteine bonds of CMP-Ang1.

### Dimeric CMP-Ang1, CA1-3, activates Tie2-Akt signaling and promotes *in vitro* angiogenic activities in cultured endothelial cells

**Figure 2:**
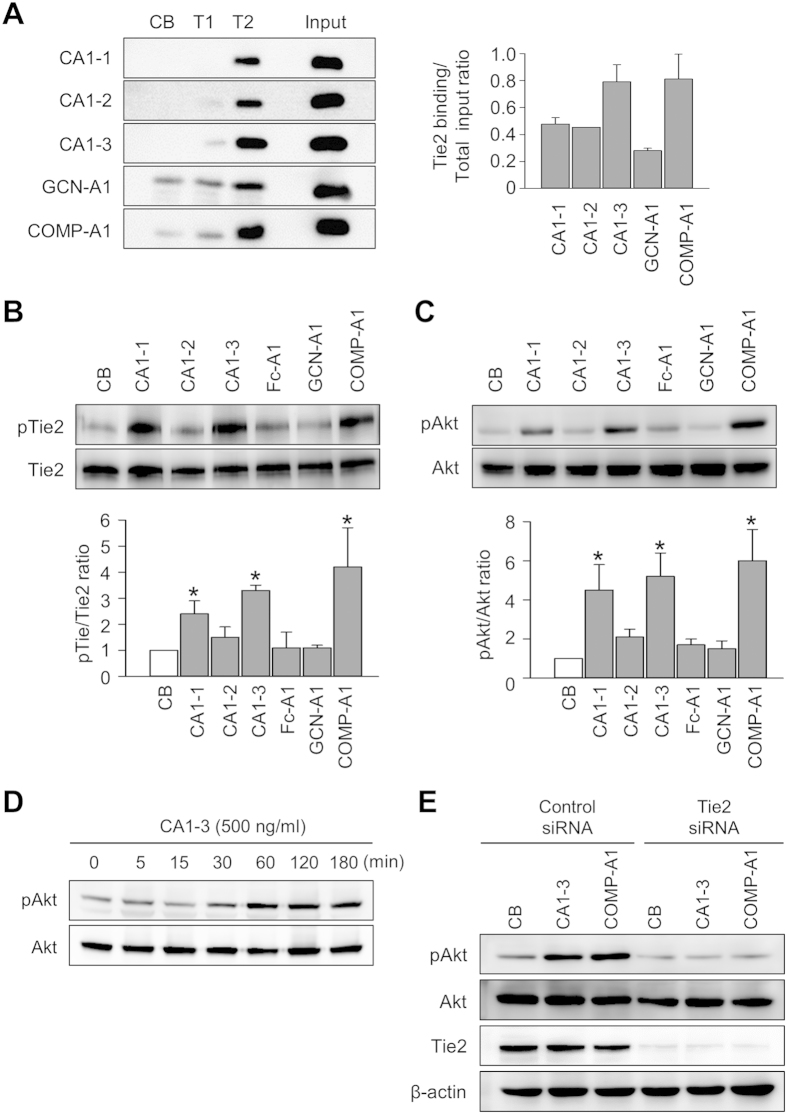
Dimeric CMP-Ang1 binds to Tie2 and activates Tie2/Akt signaling. (**A**) *In vitro* binding assay of CA1-1, CA1-2, CA1-3, GCN-Ang1, and COMP-Ang1 with control buffer (CB), Tie1-Fc (T1), or Tie2-Fc (T2). The graph shows the T2 binding ratios of each Ang1 variant over total input protein. Each graph indicates the mean ± SD from three experiments. (**B,C**) Serum-starved HUVECs were treated with either control buffer (CB), 500 ng/ml of CA1-1, CA1-2, CA1-3, Fc-Ang1, GCN-Ang1, or COMP-Ang1 for 15 min for Tie2 phosphorylation (**B**) or for 30 min for Akt phosphorylation (**C**). The relative ratio of phospho-Tie2 (pTie2) to Tie2 or phospho-Akt (pAkt) to Akt of CB is arbitrarily given as 1. Each graph represents means ± SD from three experiments. *p < 0.05 versus CB. (**D**) The Akt phosphorylation activity of CA1-3 was measured in a time-dependent manner. (**E**) HUVECs were transfected with Tie2 siRNA or control siRNA. At 24 hr after transfection of siRNA, 500 ng/ml of CA1-3 or COMP-Ang1 were added for 30 min, and then cell lysates were analyzed by western blotting using antibodies against pAkt, Akt, Tie2, or β-actin.

To compare the biochemical and biological activities of each Ang1 variants, *in vitro* binding assay was performed and the effects on Tie2 and Akt, a major component of the intracellular Tie2 signaling pathway, were measured. *In vitro* binding assays revealed that, compared with COMP-Ang1, CA1-1, CA1-2, Fc-Ang1, and GCN-Ang1 exhibited relatively lower binding to Tie2, which were similar to previous findings^9^,^11^, whereas CA1-3 unexpectedly exhibited an equivalent binding to Tie2 (Fig. 2A; Fc-Ang1 result not shown). However, none of these Ang1 variants bound significantly to Tie1 (Fig. 2A). We next examined Tie2 and Akt phosphorylation assay to compare the activities of Ang1 variants. Dimeric CA1-3 strongly phosphorylated Tie2 and Akt (Ser473), which was to a slightly higher extent than trimeric CA1-1 but slightly less than pentameric COMP-Ang1, in cultured human umbilical vein endothelial cells (HUVECs) (Fig. 2B,C). On the other hand, neither the trimeric CA1-2, dimeric Fc-Ang1, nor dimeric GCN-Ang1 induced Tie2 and Akt phosphorylation (Fig. 2B,C). Moreover, Akt phosphorylation induced by CA1-3 was persistent for 3 hr (Fig. 2D), which is similar to COMP-Ang1’s activity. To test whether the activity of CA1-3 on Akt phosphorylation is dependent on Tie2 receptor activation, we transfected HUVECs with Tie2 siRNA to deplete Tie2. Consequently, CA1-3 and COMP-Ang1 were unable to induce Akt phosphorylation in the Tie2-depleted HUVECs, while CA1-3 and COMP-Ang1 successfully induced Akt phosphorylation in the control siRNA-transfected HUVECs (Fig. 2E). Thus, CA1-3 can strongly and specifically bind and activate Tie2, and activate the main Tie2 downstream signaling, Akt, in the vascular endothelial cells despite its dimeric status.

**Figure 3:**
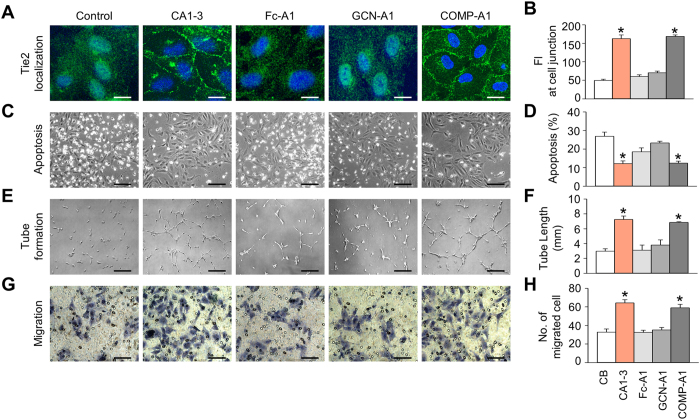
Biological effects of dimeric CMP-Ang1 in HUVECs. (**A,B**) Confluent HUVECs were starved in serum-free medium and stimulated with either control buffer (CB), 500 ng/ml of CA1-1, CA1-2, CA1-3, GCN-Ang1, FC-Ang1, or COMP-Ang1 for 15 min. (**A**) Images of HUVECs stained for Tie2 (green) and nucleus (blue). Scale bars, 10 μm. (**B**) Quantification of the fluorescence intensity (FI) of Tie2 at cell junction. (**C**,**D**) HUVECs were incubated with serum-free medium containing CB or 500 ng/ml of Ang1 variants. (**C**) Phase-contrast photographs were taken at 48 hr after serum starvation. Scale bars, 100 μm. (**D**) Apoptotic cells were measured by TUNEL immunostaining and flow cytometry. (**E**,**F**) HUVECs were plated on Matrigel^TM^ coated wells and were incubated with serum-free medium stimulated with CB or 500 ng/ml of Ang1 variants. (**E**) Phase-contrast photographs were taken at 7 hr after treatment. Scale bars, 100 μm. (**F**) Tube formation activities were measured by total tube length. (**G**,**H**) Migration activity assay was performed using a modified Boyden chamber assay. HUVECs were seeded in the upper layer of 8 μm-pore membrane. Serum-free medium containing 500 ng/ml of Ang1 variants was added in the bottom of the chamber. (**G**) The migrated cells were fixed and stained after 9 hr incubation. Scale bars, 100 μm. (**H**) The migrated cells on the bottom of membrane were counted. Each graph indicates the mean ± SD of three experiments. *p < 0.05 versus CB.

To address the effects of CA1-3 on cultured HUVECs, we compared its activities with Fc-Ang1, GCN-Ang1, and COMP-Ang1. Indeed, like COMP-Ang1^19^,^20^, CA1-3 induced Tie2 trans-localization to cell-to-cell junctions, promoted tube formation by 2.4-fold, and increased migration by 2.0-fold, while suppressing apoptosis by 2.2-fold compared with control (Fig. 3). Meanwhile, as we expected, Fc-Ang1 and GCN-Ang1 did not significantly alter the cellular activities of cultured HUVECs (Fig. 3). Thus, dimeric CMP-Ang1 is as potent as COMP-Ang1 in inducing *in vitro* activities of endothelial stabilization and angiogenesis.

### Dimeric CMP-Ang1 has different glycan patterns depending on the host cell line

**Figure 4:**
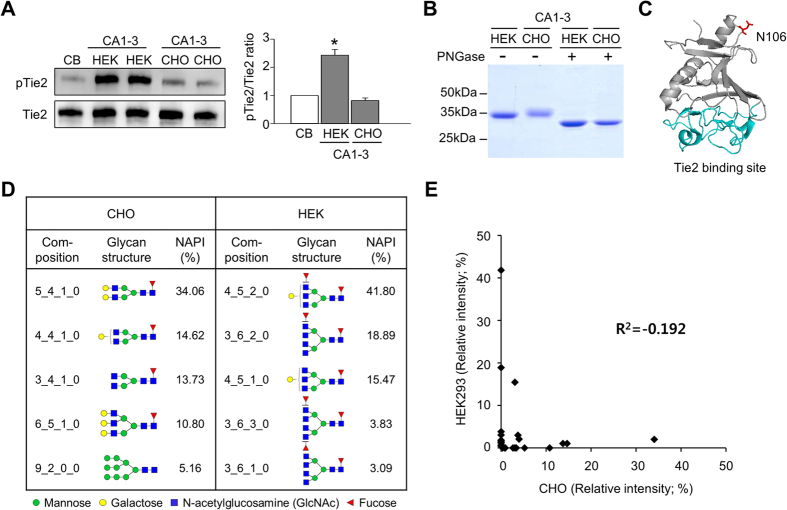
Dimeric CMP-Ang1 has different glycan pattern dependent on cell line. (**A**) Serum-starved HUVECs were treated with 500 ng/ml of CA1-3 expressed by either HEK293 or CHO cells for 15 min and then the phosphorylation of Tie2 was detected by immunoprecipitation and immunoblotting. The relative ratio of phospho-Tie2 (pTie2) to Tie2 in CB-treated cells is arbitrarily given as 1. Each graph represents the mean ± SD of three experiments. *p < 0.05 versus CB. (**B**) Analysis of CA1-3 expressed by either HEK293 or CHO cells after treatment of PNGase F to remove N-glycosylation. Reduced protein samples were separated by SDS-PAGE and were stained with Coomassie blue. (**C**) Crystal structure of FLD in Ang1. The N-glycosylation site, asparagine at 106 residue, is colored in red and the Tie2 binding site is colored in cyan. (**D**) Analysis of N-glycan of CA1-3 produced from HEK293 and CHO cells. Five glycans with the highest NAPI (normalized absolute peak intensity) values for both CHO and HEK293 cells are shown. Composition represents Hex_HexNAc_Fuc_NeuAc. (**E**) Correlation of glycans in CA1-3 between HEK293 and CHO cells. R^2^ value is indicated.

Based on the aforementioned findings, we chose CA1-3 for further biochemical analyses. For a steady and large-scale production of CA1-3, we attempted to produce CA1-3 protein using a commonly used CHO cell line (DG44)^21^. However, our assays indicated that the CA1-3 produced in CHO cells did not induce Tie2 phosphorylation, while the CA1-3 produced in HEK293 cells reproducibly induced Tie2 phosphorylation (Fig. 4A). Similar findings were also observed when we generated CA1-3 in different CHO cell line (CHO-K1)^22^ (results not shown). Because it is well-known that the glycosylation patterns of recombinant proteins produced in HEK293 is different from those produced in CHO cells, and that different glycosylation patterns can significantly alter the activity of a protein^23^,^24^, we first sought to examine the glycosylation pattern and activities of the CA1-3 produced from the two cell types.

SDS-PAGE analysis revealed that the CA1-3 produced in HEK293 cells had smaller molecular weight compared with that produced in CHO cells. To test whether this difference is associated with their glycosylation status, the proteins were treated with PNGase F to remove their N-glycosylation. After deglycosylation, the molecular weight of these two proteins decreased to the same level and their weight was no longer different, indicating that the glycosylation patterns of the two CA1-3 produced by these cell lines are different (Fig. 4B). For a more detailed glycosylation analysis, LC/MS was performed to locate the N-glycosylation site in CA1-3. It showed that CA1-3 has a single N-glycosylation site at 106 residue in the fibrinogen-like domain (Fig. 4C), which is not directly involved in Tie2 binding^15^. Next, to clarify the exact glycosylation pattern, N-glycan profiling of CA1-3 produced in both cell lines was performed by MALDI-MS. As a result, we found that the CA1-3 produced in HEK293 cells had either complex tri- (~57%) or tetra- (~25%) antennary type glycans carrying either one or two fucose residues each, whereas the CA1-3 produced in CHO cells mainly had complex bi-antennary type glycans (~62%) (Fig. 4D). Additionally, we examined the correlation of overall glycosylation using normalized peak intensity of neutral glycans. As shown in Fig. 4E, the glycans of CA1-3 from HEK293 and CHO cells did not have any correlation, indicating that glycosylation depends on host cell lines. These findings demonstrate that CA1-3 from different cell lines can have significantly different glycan complex and activity.

### N-glycosylation in dimeric CMP-Ang1 is critical for protein activity

**Figure 5:**
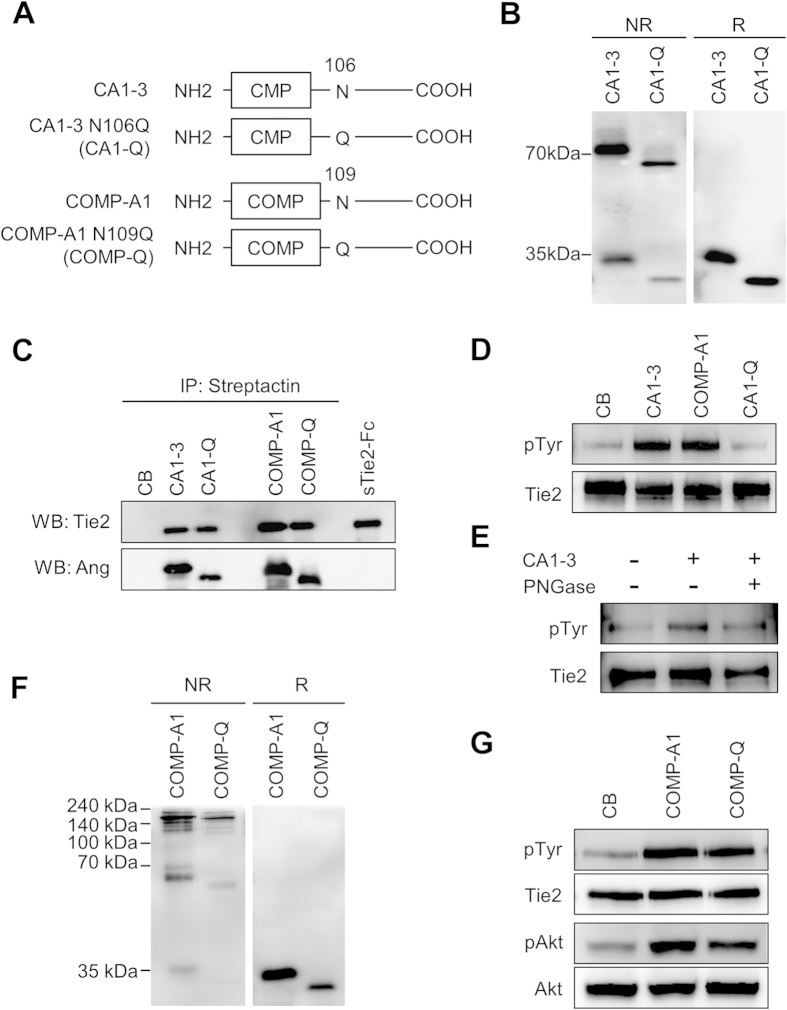
N-glycosylation of dimeric CMP-Ang1 is critical for protein activity. (**A**) Schematic diagram of N-glycosylation mutants of CA1-3 and COMP-Ang1 (CA1-Q and COMP-Q, respectively). For CA1-Q, the N-glycosylation site, asparagine (N) at 106 residue of CA1-3 is substituted to glutamine (Q) to inhibit N-glycosylation. Similarly, for COMP-Q, the N-glycosylation site at 109 residue of COMP-Ang1 is substituted to glutamine (Q). (**B**) CA1-3 and CA1-Q were separated by SDS-PAGE under non-reduced (NR) and reduced (R) conditions, and immunoblotted with anti-Ang1 antibody. Molecular masses (kDa) are indicated on the left. (**C**) *In vitro* binding assay of CA1-3, CA1-Q, COMP-Ang1, and COMP-Q to Tie2. (**D**) HUVECs were starved and then stimulated with 500 ng/ml of indicated proteins for 15 min and the phosphorylation of Tie2 was detected by immunoprecipitation and immunoblotting. (**E**) Tie2 phosphorylation after treatment with either control buffer, CA1-3, or CA1-3 and PNGase F. (**F**) SDS-PAGE analysis of COMP-Ang1 and COMP-Q. (**G**) Tie2 and Akt phosphorylation activity of COMP-Ang1 and COMP-Q.

To investigate whether the difference in the N-glycosylation pattern of CA1-3 is responsible for its activity on Tie2 phosphorylation, we replaced the amino acid at the N-glycosylation site, the asparagine at 106 residue, of CA1-3 to glutamine via a site-directed mutagenesis (CA1-Q) (Fig. 5A). The original and mutant forms were transiently expressed by HEK293 cells and the protein weights were confirmed by SDS-PAGE analysis. As expected, CA1-Q was smaller in molecular weight compared with CA1-3 in both NR and R conditions (Fig. 5B). Intriguingly, our immunoprecipitation analysis showed that although CA1-Q was able to bind to Tie2, it had completely lost its activity on Tie2 phosphorylation (Fig. 5C,D). Moreover, enzymatically deglycosylated CA1-3 also lost its Tie2 phosphorylation activity (Fig. 5E), indicating that N-glycosylation at 106 residue in dimeric CMP-Ang1 is critical for the activation of Tie2.

We next questioned whether N-glycosylation is also critical to COMP-Ang1 for its ability to activate Tie2, since COMP-Ang1 and CA1-3 both share the same fibrinogen-like domain. To test this, we generated a deglycosylated form of COMP-Ang1 (COMP-Q), which had its asparagine at the N-glycosylation site (109 residue) substituted to glutamine (Fig. 5A,F). Interestingly, COMP-Q was still capable of inducing Tie2 and Akt phosphorylation unlike CA1-Q (Fig. 5G). These findings indicated that although the N-glycosylation pattern was irrelevant to the activity of COMP-Ang1, it is crucial for CA1-3 in its ability to activate Tie2 signaling. However, it is still not clear that how N-glycan of CA1-3 is utilized for Tie2 activation.

### Dimeric CMP-Ang1 induces vascular enlargement and prevents vascular permeability *in vivo*

**Figure 6:**
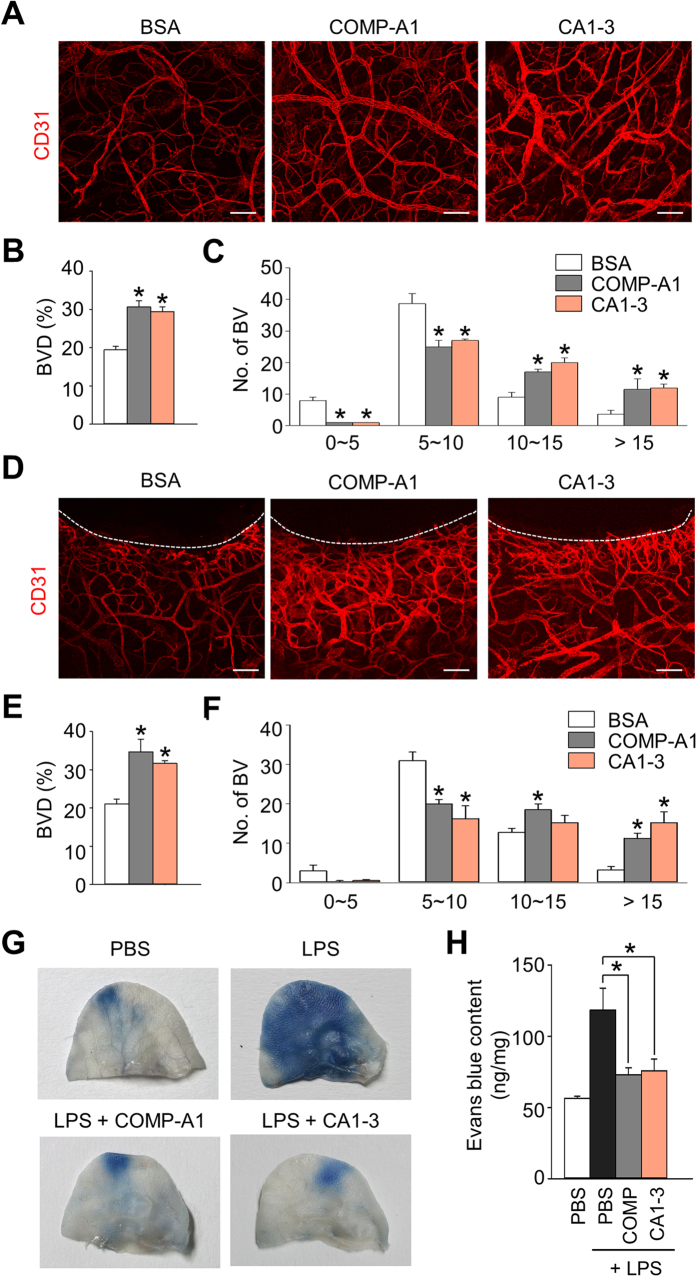
Dimeric CMP-Ang1 induces angiogenesis and prevents vascular permeability *in vivo*. (**A–F**) One μg of BSA, CA1-3 and COMP-Ang1 were intradermally injected to normal ears (**A–C**) and hole-punch injury ears (**D–F**) every 12 hr for 7 days. Blood vessels in normal ear skin (**A**) and wound margin of ear skin (**D**) were visualized by CD31 immunostaining. Blood vessel densities (BVD) (**B**,**E**) and numbers of different-sized vessels (**C**,**F**) in the normal and wound margin of ear skin were measured. Each graph indicates the mean ± SD of five experiments. Scale bars, 100 μm. *p < 0.05 versus BSA. (**G**,**H**) Ears were treated intradermally with PBS, LPS, or LPS with CA1-3 or COMP-Ang1 and then Evans blue dye was injected intravenously to visualize plasma leakage. Gross images of Evans blue dye extravasation in ears (**G**) and its quantification (**H**). Each graph indicates the mean ± SD of four experiments. *p < 0.05 versus LPS-treated group.

Vascular enlargement is one of the hallmarks of Ang1-induced vascular remodeling in quiescent normal vessels^25^. To examine the effect of CA1-3 *in vivo*, we treated the recombinant protein to a normal ear every 12 hr. After 7days of treatment, CA1-3 and COMP-Ang1 both induced vessel enlargement, markedly increasing the number of large-sized vessels (>10 μm), and increased blood vessel densities by 1.6- and 1.5-folds, respectively, in normal ear skin compared with those treated with bovine serum albumin (BSA) (Fig. 6A–C).

Next, to evaluate the effect of CA1-3 in blood vessel remodeling during wound healing *in vivo*, we used a hole-punch injury model with the ears of adult mice and treated them with the recombinant proteins every 12 hr. After 7 days of treatment, more enlarged and denser blood vessels were visible, mostly at the margin of the wound healing area, in the ears of both the COMP-Ang1 and CA1-3-treated mice compared with BSA-treated mice (Fig. 6D). The blood vessel densities exhibited 1.6- and 1.5-fold increases by COMP-Ang1 and CA1-3, respectively (Fig. 6E), and the COMP-Ang1 and CA1-3-treated mice had more enlarged vessels (>10 μm) than BSA treated mice (Fig. 6F).

Ang1 also known to stabilize blood vessel and prevents vascular leakage in several inflammatory settings^26^,^27^. To determine whether CA1-3 could also protect blood vessels from vascular leakage, we measured Evans blue extravasation in the ear skin after topical challenge with LPS either with or without COMP-Ang1 or CA1-3. While the treatment of LPS alone resulted in a marked increase of dye extravasation into the ear, the treatment of LPS in combination with either CA1-3 or COMP Ang1 successfully blocked the vascular leakage induced by LPS (Fig. 6G,H). Taken together, we confirmed that CA1-3 effectively induces angiogenesis and suppresses vascular leakage *in vivo*.

## Discussion

In this study, we designed and generated a dimeric CMP-Ang1 variant, which has a modified form of the coiled-coil domain of CMP integrated into native Ang1, using mammalian cell expression system. Throughout SDS-PAGE, TEM, *in vitro*, and *in vivo* biological assays, we provide a critical clue about the potency and usefulness of dimeric CMP-Ang1 as an agonist of Tie2 receptor.

Unlike other receptor tyrosine kinases, Tie2 receptors interact with highly oligomeric Ang1, leading to receptor oligomerization and activation without major conformational change in its extracellular region^14^,^15^. Although it has not been fully understood as to how the oligomerization of Tie2 upon ligand binding leads to receptor activation, Ang1-induced Tie2 oligomerization is a critical process for activating Tie2 receptor and initiating its downstream signaling. Previous studies demonstrated that engineered dimers of Ang1, Ang1-F1-Fc^9^ and GCN-Ang1^11^, were able to bind to Tie2 but were insufficient to phosphorylate Tie2, suggesting that those dimeric Ang1 variants could not promote receptor oligomerization for Tie2 activation. However, Anisimov *et al.* recently generated a VEGF-angiopoietin-1(VA1) chimeric protein, which formed disulfide-bonded dimers^28^. Despite its dimeric structure, VA1 activated Tie2 and ameliorated vascular leakage and inflammation that were induced by VEGF-A while enhancing vessel diameter for better perfusion, which were all comparable to the effects of oligomeric Ang1. Their findings are basically consistent with our results with CA1-3. Our study showed that CA1-3 not only had superior ability to activate its receptor over any other dimeric Ang1 variants but was also more potent than other higher oligomeric CMP-Ang1 mutants. Furthermore, CA1-3 phosphorylated Tie2 to a comparable level to that of COMP- Ang1 and similarly promoted both *in vitro* and *in vivo* angiogenesis. Hence, our study suggests that dimeric oligomers of Ang1 could be sufficient to induce proper oligomerization of Tie2 for receptor activation.

We questioned how dimeric CMP-Ang1 is able to activate Tie2. Our analyses indicated that the glycosylation pattern in CA1-3 influences Tie2 activation. While the CA1-3 produced by HEK293 cells strongly activated Tie2, the CA1-3 produced by CHO cells could not induce Tie2 activation. It is well-known that that glycosylation affects protein characteristics ranging from folding, stability, and protein interaction to more physiological properties such as protein activity^29^,^30^,^31^, and various factors influence protein glycosylation patterns, including protein amino acid sequence, the host cell in which recombinant proteins are expressed, and growth conditions. With regards to host cell-specific effects on glycosylation, we observed significant differences in N-glycosylation of CA1-3 depending on whether it was produced by HEK293 or CHO cells. SDS-PAGE analysis revealed that the protein band of CHO-derived CA1-3 was more diffused than that of HEK293-derived CA1-3, which is an indication of a more heterogeneous mass distribution. We also found that most of N-glycan structures on the Asn-106 site of CA1-3 produced by HEK293 cells are either highly branched tri- or tetra-antennary types, while those expressed by CHO mainly have bi-antennary types; such difference might be linked to the changes in the protein activity. Interestingly, although the N-glycan site exists outside the receptor-binding site, the protein activity was almost completely lost by a mutation of the N-glycan site in CA1-3. Thus we hypothesize that the N-glycosylated site at Asn-106 in CA1-3 is not directly involved in the binding ability to Tie2, but may instead be associated with the ability to rearrange Tie2 to cell-to-cell junctions and activate Tie2 and its subsequent cellular signaling.

Thus far, CHO cell system has been the most widely used cell line for the production of therapeutic proteins due to their capacity for high-level production and human-like glycosylation. However, CHO cells cannot produce all types of human glycan structures as they lack several glycosylation enzymes present in human cells. Thus, the biological activities of the recombinant proteins produced by CHO cells can be significantly different to those produced by human cell lines due to the altered glycan profiles. In addition, CHO cells generate therapeutic proteins with potentially immunogenic glycan structures^32^. For these limitations, it is advantageous and sometimes essential to produce certain recombinant proteins in human cells such as HEK293 cells. Human cells are expected to produce recombinant proteins with the same glycan profiles as natural proteins. One example is Xigris (activated protein C), which is produced in HEK293 cells since the post-transitional modifications for maintaining its biological activity were not adequate in CHO cells^32^,^33^. Thus, choosing an expression system for production of glycoproteins with appropriate structures for their function should be carefully assessed.

Questions remain about how GCN-Ang1 and Fc-Ang1 could not activate Tie2, while dimeric CMP-Ang1 could. To address this question, we examined whether glycosylation or relative flexibility of FLD on GCN-Ang1 and Fc-Ang1 would affect Tie2 activation. Contrary to dimeric CMP-Ang1, glycosylation or relative flexibility of FLD did not alter the activity of GCN-Ang1 and Fc Ang1 (results not shown). Although it is not clear how dimeric CMP-Ang1 can activate Tie2 and what makes the difference in Tie2 activation between dimeric CMP-Ang1 versus GCN-Ang1 or Fc-Ang1, our results suggest that certain types of glycan on dimeric CMP-Ang1 is absolutely required to activate Tie2. Moreover, the proper orientation or location of FLD of Ang1 which is altered by the domain tethering FLD of Ang1 could be critical in activating Tie2.

In conclusion, our findings indicate that dimeric CMP-Ang1 is sufficient to activate Tie2, but this activity is dependent on the specific N-glycosylation within the fibrinogen-like domain. The dimeric CMP-Ang1 strongly bound to and activated Tie2 to a comparable level to that of COMP-Ang1. It also had a similar potency as COMP-Ang1 in promoting endothelial survival, migration, and tube formation *in vitro*, as well as in increasing adult angiogenesis and inhibiting vascular permeability *in vivo*. Our study indicates that dimeric CMP-Ang1 could lead to adequate Tie2 activation. However, further investigation is required to elucidate the exact mechanism behind the enigma to Tie2 clustering and activation upon binding of dimeric CMP-Ang1 to Tie2.

## Methods

### Gene constructs

pcDNA vector (Invitrogen) that contains a secretory signal sequence for hemagglutinin and a Strep-tagII (WSHPQFEK) was used to express the linker and fibrinogen-like domain of Ang1 which is fused to the coiled-coil domain of CMP, GCN4, or COMP or the Fc portion of immunoglobulin. CA1-2 and CA1-3 were generated by substituting the cysteine at 76 residue and the cysteines at 31 and 76 residues, respectively, to alanine by site-directed mutagenesis using CA1-1 as a template. N-glycosylation mutants were generated by site-directed mutagenesis using CA1-3 or COMP-Ang1 construct as a template.

### Transfection and purification of recombinant proteins

The recombinant proteins were transiently expressed in human embryo kidney 293 (HEK293) cells (CRL-10852, ATCC) using Jet-PEI^TM^ (Polyplus-transfection Inc.) or dhfr-deficient CHO (DG44) cells (CRL-9096, ATCC) using Lipofectamin 2000 (Invitrogen). DG44 and HEK293 cells were purchased from indicated manufacturers who certified a contamination of virus and microbe, and cell performances. These have been cultured for fewer than 6 months after receipt. The transfected HEK293 cells and DG44 cells were maintained as previously reported^18^,^34^. The secreted recombinant proteins in cultured media were purified by column chromatography with streptactin sepharose affinity gel (IBA) and eluted with D-desthiobiotin (Sigma-Aldrich). After purification, the concentration of recombinant proteins were measured by Bradford assay and confirmed by Coomassie blue staining of an SDS-polyacrylamide gel.

### Transmission Electron microscopy

Two μl of purified proteins were applied to glow-discharged copper grids with continuous carbon film and negatively stained with 0.75% (w/v) uranyl formate for 30 sec^35^,^36^. The EM grids were examined under a Tecnai T120 microscope (FEI Company) operated at 120 kV. Images were recorded at a nominal magnification of 67,000x on an FEI Eagle 4 K × 4 K charge-coupled device (CCD) camera (1.64 Å/pixel). Defocuses were between −1.0 μm and −1.5 μm and electron dose was ~30 e-/Å^2^.

### Characterization of recombinant proteins and *in vitro* binding assay

The media of transfected HEK293 cell were immunoprecipitated with streptactin sepharose and were separated by SDS-PAGE under reducing (R) and non-reducing (NR), followed by immunoblotting with anti-Ang1 antibody (sc-8357, Santa Cruz). Binding of the recombinant proteins to the soluble extracellular domain of Tie1-Fc (sTie1-Fc, T1) or Tie2-Fc (sTie2-Fc, T2) (R&D systems) was assayed using an *in vitro* binding assay; 100 ng of each recombinant protein was mixed with 500 ng of sTie1-Fc or sTie2-Fc and incubated in 500 μl of Tris-buffer solution with NP-40 (TBSN; 50 mM Tris, 100 mM NaCl, 1.0% Nonidet P-40, pH 7.5) at 4 °C for 2 hr. Protein-A agarose beads (Oncogene) or streptactin agarose beads were added and incubated at 4 °C for 1 hr. The samples were eluted with sample buffer and heat-denatured under R conditions. The samples were separated by SDS-PAGE, transferred onto nitrocellulose membrane, and immunoblotted with horseradish-peroxidase (HRP)-conjugated streptactin (Bio-rad), anti-human IgG antibody or anti-Ang1 antibody. All signals were visualized by LAS-4000 (Fuji Film).

### Size-exclusion chromatography

Size-exclusion chromatography was performed by FPLC using a Superdex 75 10/300 GL column (GE Healthcare) equilibrated with 100 mM Tris-Cl (pH 8.0) buffer containing 150 mM NaCl.

### Tie2/Akt phosphorylation

Human umbilical vein endothelial cells (HUVECs) were grown in EGM-2 (Lonza) with 2% FBS. The primary cultured HUVECs used for this study were within 7 passages. Confluent cells were starved for 12 hr and were treated with indicated proteins for 15 min (for Tie2 phosphorylation) or 30 min (for Akt phosphorylation). For Tie2 phosphorylation, the cell lysates were immunoprecipitated with anti-Tie2 antibody (R&D) and immunoblotted with anti-phosphotyrosine antibody (4G10, Millipore). For Akt phosphorylation, 50 μg of cell lysates were loaded in SDS-PAGE and immunoblotted with anti-phospho-Akt antibody (Cell Signaling). The membranes were stripped and reprobed with anti-Tie2 antibody (Santa Cruz) or anti-Akt antibody (Cell Signaling) to verify equal amount of protein. All signals were visualized by LAS-4000 (Fuji Film).

### RNA interference

Control small interfering RNA (siRNA) or siRNA directed to human Tie2 was transfected using Lipofectamin RNAi max (Invitrogen). The cells were analyzed 24 hr after transfection. Down-regulation of Tie2 was verified by immunoblotting.

### Tie2 localization

Confluent HUVECs were starved for 12 hr and the cells were treated with indicated proteins. After 15 min, the cells were fixed with 4% formaldehyde and permeabilized with 0.5% Triton X-100. The cells were blocked with PBS containing 5% donkey serum for 1 hr and immunostained with anti-Tie2 (sc-324, Santa Cruz) overnight. After several washes with PBS containing 0.1% Triton X-100, the cells were incubated with 1 hr with cy3-conjugated secondary antibodies. Fluorescent signals were visualized and digital images were obtained using Zeiss LSM 780 confocal microscope (Carl Zeiss).

### Tube formation

For tube formation assay, 5 × 10^4^ cells of HUVECs were seeded on Matrigel^TM^ (BD Biosciences)-coated 4-well plates and treated with indicated proteins. After 9 hr incubation, the cells were photographed, and tube-like structure lengths were measured to quantify tube forming activities.

### Survival assay

Confluent HUVECs were incubated with indicated protein in serum free media for 48 hr. The cells were photographed using a phase-contrast microscope, then the cells was analyzed by the TdT-mediated dUTP nick-end Labeling (TUNEL) assay kit (Roche).

### Migration assay

Migration assay was performed using a modified Boyden chamber with polycarbonate filter (8 μm pores, Corning) coated with 0.1% gelatin; 1 × 10^5^ HUVECs were seeded in the upper chamber and the indicated concentration of the recombinant protein in serum-free medium was added in the bottom chamber. After 6 hr incubation, the non-migrated cells were removed from the upper side of the filter with a cotton swab. The migrated cells were fixed with 4% formaldehyde and stained with crystal violet (Sigma-Aldrich). The migrated cells were counted at 400x magnification using a microscope.

### Glycan analysis by mass spectrometry

N-glycan release and associated processing steps were performed according to previously optimized procedures^37^. Briefly, glycoproteins were thermally denatured in an aqueous solution of ammonium bicarbonate and dithiothreitol. Glycans were enzymatically released using peptide N-glycosidase F (New England Biolabs, Beverly, MA) in a water bath at 37°C for 16 hr. Released N-glycans were purified by solid phase extraction using graphitized carbon cartridges (Grace Davison, Deerfield, USA). An UltrafleXtreme MALDI-TOF/TOF instrument (Bruker Daltonik GmbH) equipped with a smartbeam II laser (Nd: YAG, 355 nm) was used for glycan profiling in reflectron ion mode. 2,5-Dihydroxy-benzoic acid was used as a matrix (5 mg/100 ml in 50% Acetonitrile) and a saturated solution of NaCl was used as a cation dopant to increase signal sensitivity only in positive mode. Raw MS data was processed with flexAnalysis software (version 3.3, Bruker Daltonics, MA).

### Statement of ethics of animal care and use

Animal care and experimental procedures were performed under the approval from the Animal Care Committees of KAIST. All animal procedures were carried out in accordance with KAIST guideline for Animal Experimentation and the National Institutes of Health guidelines, including “Principles for Use of Animals” and “Guide for the Care and Use of Laboratory Animals”.

### *In vivo* angiogenesis assay

Animal care and experimental procedures were performed under the approval from the Animal Care Committees of KAIST. Specific pathogen-free FVB/N mice were purchased from Jackson Laboratory and bred in our pathogen-free animal facility. Male 8-week-old mice were used for this study. For hole-punch assays, a 2.0 mm hole was made in one ear of mouse using a metal ear punch (Harvard Apparatus, Holliston). One μg of BSA, CMP-A1-3, and COMP-Ang1 dissolved in 10 μl of sterile 0.9% NaCl was intradermally injected in normal and wounded ears every 12 hr. At 7 days later, the mice were anesthetized with 80 mg/kg of ketamine hydrochloride and 12 mg/kg of xylazine and the ear skins were harvested and immunostained as whole mounts. After blocking with 5% donkey serum in PBST (0.3% Triton X-100 in PBS) for 1 hr, samples were incubated with anti-mouse PECAM-1 antibody (hamster clone 2H8, 1:1000) for 6 hr. After several washes in PBST, the samples were incubated for 4 hr with FITC-conjugated anti-hamster IgG antibody (1:1000). Fluorescent signals were visualized and digital images were obtained using a Zeiss LSM 780 confocal microscope (Carl Zeiss). Blood vessels densities and vessel diameter were measured using the image analysis software.

### Vascular permeability assay

Vascular permeability was quantified using the Miles assay. LPS alone (1 μg) or recombinant protein (1 μg) with LPS in 10 μl of PBS was intradermally injected in the ear and then Evans blue dye was injected intravenously into mice. Ears were collected 30 min after Evans blue injection and incubated with formamide at 56 °C for 48 hr. The concentration of Evans blue in ear and standards was determined by reading the optical density at 620 nm.

### Statistical analysis

Values are presented as mean ± standard deviation. Statistical differences between means were determined by Mann-Whitney test. Statistical significance was set at p < 0.05.

## Additional Information

**How to cite this article**: Oh, N. *et al.* A Designed Angiopoietin-1 Variant, Dimeric CMP-Ang1 Activates Tie2 and Stimulates Angiogenesis and Vascular Stabilization in N-glycan Dependent Manner. *Sci. Rep.* **5**, 15291; doi: 10.1038/srep15291 (2015).

## Figures and Tables

**Table 1:** Biochemical characteristics of Ang1 variants.

	Elution, %	Solubility, %	Stability, %	Aggregation, %
CA1-1	> 90	> 90	> 95	Not detectable
CA1-2	> 90	> 90	ND	Not detectable
CA1-3	> 90	> 90	> 95	Not detectable
Fc-A1	> 90	> 90	ND	Not detectable
GCN-A1	> 85	> 90	ND	Not detectable
COMP-A1	> 90	> 90	> 95	> 10%
Native Ang1	> 65	> 70	> 60	> 50%

Elution is the percentage of protein released from streptactin agarose beads. Solubility is the percentage of soluble protein at pH 4.0 in acetic acid buffer. Insoluble proteins were precipitated. Elution and solubility were measured with freshly prepared proteins. Stability is the phosphorylation activity of proteins on Tie2 in HUVECs after being stored at −70° C for 3 months. Aggregation is the percentage of protein aggregates under non-reducing condition in the stacking gel of SDS-PAGE after being stored at −70° C for a month. Three repeats of each experiment showed similar findings. ‘ND’ is ‘not determined’.

## References

[b1] Davis S. *et al.* Isolation of angiopoietin-1, a ligand for the TIE2 receptor, by secretion-trap expression cloning. Cell 87, 1161–1169 (1996). 8980223 10.1016/s0092-8674(00)81812-7

[b2] Augustin H. G., Koh G. Y., Thurston G. & Alitalo K. Control of vascular morphogenesis and homeostasis through the angiopoietin–Tie system. Nat. Rev. Mol. Cell Bio. 10, 165–177 (2009). 19234476 10.1038/nrm2639

[b3] Koh G. Y. Orchestral actions of angiopoietin-1 in vascular regeneration. Trends Mol. Med. 19, 31–39 (2013). 23182855 10.1016/j.molmed.2012.10.010

[b4] Thurston G. *et al.* Leakage-resistant blood vessels in mice transgenically overexpressing angiopoietin-1. Science 286, 2511–2514 (1999). 10617467 10.1126/science.286.5449.2511

[b5] Suri C. *et al.* Requisite role of angiopoietin-1, a ligand for the TIE2 receptor, during embryonic angiogenesis. Cell 87, 1171–1180 (1996). 8980224 10.1016/s0092-8674(00)81813-9

[b6] Arita Y. *et al.* Myocardium-derived angiopoietin-1 is essential for coronary vein formation in the developing heart. Nat. Commun. 5, 10.1038/ncomms5552 (2014). PMC4124867 25072663

[b7] Jeansson M. *et al.* Angiopoietin-1 is essential in mouse vasculature during development and in response to injury. J. Clin. Invest. 121, 2278 (2011). 21606590 10.1172/JCI46322 PMC3104773

[b8] Procopio W. N., Pelavin P. I., Lee W. M. & Yeilding N. M. Angiopoietin-1 and-2 coiled coil domains mediate distinct homo-oligomerization patterns, but fibrinogen-like domains mediate ligand activity. J. Biol. Chem. 274, 30196–30201 (1999). 10514510 10.1074/jbc.274.42.30196

[b9] Davis S. *et al.* Angiopoietins have distinct modular domains essential for receptor binding, dimerization and superclustering. Nat. Struct. Mol. Biol. 10, 38–44 (2002). 10.1038/nsb880 12469114

[b10] Kim K.-T. *et al.* Oligomerization and multimerization are critical for angiopoietin-1 to bind and phosphorylate Tie2. J. Biol. Chem. 280, 20126–20131 (2005). 15769741 10.1074/jbc.M500292200

[b11] Cho C.-H. *et al.* COMP-Ang1: a designed angiopoietin-1 variant with nonleaky angiogenic activity. Proc. Natl. Acad. Sci. USA. 101, 5547–5552 (2004). 15060279 10.1073/pnas.0307574101 PMC397420

[b12] Maisonpierre P. C. *et al.* Angiopoietin-2, a natural antagonist for Tie2 that disrupts *in vivo* angiogenesis. Science 277, 55–60 (1997). 9204896 10.1126/science.277.5322.55

[b13] Cho C.-H. *et al.* Designed angiopoietin-1 variant, COMP-Ang1, protects against radiation-induced endothelial cell apoptosis. Proc. Natl. Acad. Sci. USA. 101, 5553–5558 (2004). 15060280 10.1073/pnas.0307575101 PMC397421

[b14] Barton W. A. *et al.* Crystal structures of the Tie2 receptor ectodomain and the angiopoietin-2–Tie2 complex. Nat. Struct. Mol. Biol. 13, 524–532 (2006). 16732286 10.1038/nsmb1101

[b15] Yu X. *et al.* Structural basis for angiopoietin-1–mediated signaling initiation. Proc. Natl. Acad. Sci. USA. 110, 7205–7210 (2013). 23592718 10.1073/pnas.1216890110 PMC3645508

[b16] Dames S. A., Kammerer R. A., Wiltscheck R., Engel J. & Alexandrescu A. T. NMR structure of a parallel homotrimeric coiled coil. Nat. Struct. Mol. Biol. 5, 687–691 (1998). 10.1038/90444 9699631

[b17] Haudenschild D. R., Tondravi M. M., Hofer U., Chen Q. & Goetinck P. F. The Role of Coiled-coil α-Helices and Disulfide Bonds in the Assembly and Stabilization of Cartilage Matrix Protein Subunits. J. Biol. Chem. 270, 23150–23154 (1995). 7559460 10.1074/jbc.270.39.23150

[b18] Kim H.-Z., Jung K., Kim H. M., Cheng Y. & Koh G. Y. A designed angiopoietin-2 variant, pentameric COMP-Ang2, strongly activates Tie2 receptor and stimulates angiogenesis. BBA-Mole. Cell Res. 1793, 772–780 (2009). 10.1016/j.bbamcr.2009.01.018 19339208

[b19] Fukuhara S. *et al.* Differential function of Tie2 at cell–cell contacts and cell–substratum contacts regulated by angiopoietin-1. Nat. Cell Biol. 10, 513–526 (2008). 18425120 10.1038/ncb1714

[b20] Saharinen P. *et al.* Angiopoietins assemble distinct Tie2 signalling complexes in endothelial cell–cell and cell–matrix contacts. Nat. Cell Biol. 10, 527–537 (2008). 18425119 10.1038/ncb1715

[b21] Hwang S.-J. *et al.* Expression and purification of recombinant human angiopoietin-2 produced in Chinese hamster ovary cells. Protein Expres. Purif. 39, 175–183 (2005). 10.1016/j.pep.2004.09.005 15642468

[b22] Link T. *et al.* Bioprocess development for the production of a recombinant MUC1 fusion protein expressed by CHO-K1 cells in protein-free medium. J. Biotechnol. 110, 51–62 (2004). 15099905 10.1016/j.jbiotec.2003.12.008

[b23] Brooks S. A. Protein glycosylation in diverse cell systems: implications for modification and analysis of recombinant proteins. Expert Rev. Proteomic. 3, 345–359 (2006). 10.1586/14789450.3.3.345 16771706

[b24] Croset A. *et al.* Differences in the glycosylation of recombinant proteins expressed in HEK and CHO cells. J. Biotechnol. 161, 336–348 (2012). 22814405 10.1016/j.jbiotec.2012.06.038

[b25] Cho C.-H. *et al.* Long-term and sustained COMP-Ang1 induces long-lasting vascular enlargement and enhanced blood flow. Circ. Res. 97, 86–94 (2005). 15961719 10.1161/01.RES.0000174093.64855.a6

[b26] Thurston G. *et al.* Angiopoietin-1 protects the adult vasculature against plasma leakage. Nat. Med. 6, 460–463 (2000). 10742156 10.1038/74725

[b27] Mammoto T. *et al.* Angiopoietin-1 requires p190 RhoGAP to protect against vascular leakage *in vivo*. J. Biol. Chem. 282, 23910–23918 (2007). 17562701 10.1074/jbc.M702169200

[b28] Anisimov A. *et al.* Vascular endothelial growth factor-angiopoietin chimera with improved properties for therapeutic angiogenesis. Circulation 127, 424–434 (2013). 23357661 10.1161/CIRCULATIONAHA.112.127472

[b29] Elliott S. *et al.* Enhancement of therapeutic protein *in vivo* activities through glycoengineering. Nat. Biotechnol. 21, 414–421 (2003). 12612588 10.1038/nbt799

[b30] Lee J.-E. *et al.* Novel Glycosylated VEGF Decoy Receptor Fusion Protein, VEGF-Grab, Efficiently Suppresses Tumor Angiogenesis and Progression. Mol. Cancer Ther. 14, 470–479 (2014). 25534360 10.1158/1535-7163.MCT-14-0968-T

[b31] Straumann N., Wind A., Leuenberger T. & Wallimann T. Effects of N-linked glycosylation on the creatine transporter. Biochem. J. 393, 459–469 (2006). 16167890 10.1042/BJ20050857 PMC1360696

[b32] Dietmair S. *et al.* A multi-omics analysis of recombinant protein production in Hek293 cells. PLoS one 7, e43394 (2012). 22937046 10.1371/journal.pone.0043394 PMC3427347

[b33] Butler M. & Spearman M. The choice of mammalian cell host and possibilities for glycosylation engineering. Curr. Opin. Biotech. 30, 107–112 (2014). 25005678 10.1016/j.copbio.2014.06.010

[b34] Kim S. H. & Lee G. M. Functional expression of human pyruvate carboxylase for reduced lactic acid formation of Chinese hamster ovary cells (DG44). Appl. Microbiol. Biot. 76, 659–665 (2007). 10.1007/s00253-007-1041-6 17583807

[b35] Booth D. S., Avila-Sakar A. & Cheng Y. Visualizing proteins and macromolecular complexes by negative stain EM: from grid preparation to image acquisition. J. Vis. Exp. 58, 10.3791/3227 (2011). PMC3369646 22215030

[b36] Um J. W. *et al.* Structural basis for LAR-RPTP/Slitrk complex-mediated synaptic adhesion. Nat. Commun. 5, 10.1038/ncommas6423 (2014). 25394468

[b37] Oh M. J. *et al.* Analytical platform for glycomic characterization of recombinant erythropoietin biotherapeutics and biosimilars by MS. Bioanalysis 5, 545–559 (2013). 23425271 10.4155/bio.12.327

